# Combined laparoscopic Roux-en-Y gastric bypass reversal and gastric banding to treat severe hyperinsulinemic hypoglycemia: A case report and surgical video

**DOI:** 10.1016/j.ijscr.2022.107271

**Published:** 2022-06-08

**Authors:** F.-X. Terryn, B. Majerus

**Affiliations:** a Europe Hospitals – Sainte Elisabeth, Brussels, Belgium; b Clinique Saint Pierre, Ottignies, Belgium

**Keywords:** Roux-en-Y gastric bypass, Reversal, Laparoscopic, Nesidioblastosis, Hypoglycemia, Case report

## Abstract

• Non insulinoma pancreatogenous hypoglycemia syndrome is a recently described complication of Roux-en-Y gastric bypass
• Currently, there is no Gold Standard treatment.
• In this case, we successfully performed a combined laparoscopic reversal of gastric bypass and gastric banding

## Abstract (fr)

**Introduction:**

Non insulinoma pancreatogenous hypoglycemia syndrome (NIPHS) is a recently described complication of Roux-en-Y gastric bypass (RYGB) characterised by pathologic features of nesidioblastosis and hyperinsulinemic hypoglycemias that can occur months to years after surgery. Increased incretin production appears to be one of the involved mechanisms. Currently, there is no Gold Standard treatment.

**Presentation of case:**

We present the case of a 57-year-old woman with a history of silastic ring vertical gastroplasty (SRVG) converted into RYGB who suffered from severe daily episodes of neuroglycopenia due to NIPHS, with poor response to conservative and medical treatments.

At first, as it is suggested in recent literature, we decided to evaluate whether reversal would efficiently reduce episodes of hypoglycemia by attempting gastrostomy tubing of the remaining stomach. Since gastrostomy proved to be efficient, we reversed the RYGB laparoscopically and added a gastric banding to keep control over the patient's weight.

After an asymptomatic period of two months, mild hypoglycemias recurred, on average once every three weeks, easily managed by sugar ingestion. A preexisting gastroparesis was worsened by the intervention, possibly caused by vagus nerve injury secondary to the multiple gastric procedures. A year later, the patient returned to a regular lifestyle. Her weight gain was limited to 8 kg thanks to appropriate adjustments of the gastric band.

**Discussion:**

Treatment of NIPHS includes appropriate diet, pharmacotherapy and surgical procedures; partial or total pancreatectomy, laparoscopic reversal of RYGB (with or without sleeve gastrectomy) and gastric banding have been proposed. This is the first described case of severe NIPHS after RYGB successfully treated with combined laparoscopic reversal of gastric bypass and gastric banding to avoid weight regain.

**Conclusions:**

We expect this novel approach will help to overcome hypoglycemic episodes from NIPHS, while maintaining weight control and preventing the resurgence of obesity-related comorbidities. Further studies with long term follow-up are necessary.

## 1 Introduction and importance

Roux-en-Y gastric bypass (RYGB) surgery has remarkable effects on weight loss and diabetes remission and is currently the most performed bariatric procedure worldwide [1].

A well described late complication that may occur after RYGB is postprandial hypoglycemia.

Based on the first studies of self-reporting hypoglycemic episodes or hospital admissions, the prevalence was previously estimated around 0,1 % [2]. More recent publications with glucose tolerance tests or continuous glucose monitoring show a much higher prevalence, ranging from 10 to 70 % [3], [4].

Although most of these episodes can be managed with proper dietary measures, a few patients who underwent RYGB develop severe and refractory hypoglycemia episodes that may occur several times a day, with huge impact on their quality of life and which may even be life-threatening.

This case report is compliant with the SCARE 2020 guidelines [5].

## 2 Presentation of case

We describe the case of a 57-year-old woman without relevant medical history except for morbid obesity. The patient has a history of SRVG converted to RYGB requiring a gastrojejunal anastomosis redo due to perforation following endoscopic dilatation and suffered from late postprandial episodes of hypoglycemia. These severe episodes of neuroglycopenia occurred 90 to 120 min postprandial and never during the night or on an empty stomach. Medication such as acarbose and octreotid were tried but not well tolerated and did successfully prevent the recurrence of hypoglycemia. Up to 3 injections of Glucagon in one year were needed to treat the most severe episodes. This situation had a major impact on the quality of life of the patient.

A full workup excluded an insulinoma (including serum insulin and C-peptide monitoring, fasting test and pancreatic MRI) and the diagnosis of nesidioblastosis was established.

Thus, as the dietary measures and medical treatment were insufficient, we decided to perform gastrostomy tubing of the excluded stomach to assess if a conversion to original anatomy would be efficient to reduce the recurrence and severity of the hypoglycemic episodes. The patient did not suffer from any hypoglycemia during a 7-days hospital stay under glycemic monitoring. So, two weeks later, we laparoscopically reversed the RYGB and added a gastric banding to prevent weight regain. The intervention was performed by Bernard Majerus.

We provide a commented surgical video to describe this surgical procedure.

**Fig. 1:**
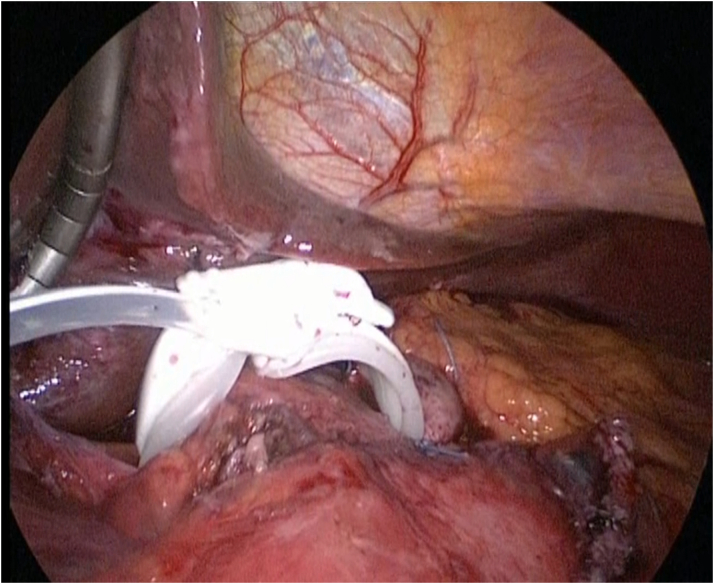
Peroperative picture after reversal of RYGB and placement of gastric banding.

At first, after a limited adhesiolysis, we removed the gastrostomy. As the different limbs were large enough, we performed linear stapling on the jejunojenunal anastomosis and kept the biliary limb in its original state. After resection of the gastrojejunal anastomosis, the digestive continuity was restored with a linear stapled side-to-side anastomosis between the biliary limb and the upper part of the alimentary limb. The previous SRVG did not allow an easy linear stapling between the two parts of the stomach. Thus, the former gastrotomy in the gastric remnant was used to perform the gastrogastrostomy with a 25 mm diameter circular stapling. The gastrostomy was stapled as well. The operation was well tolerated by the patient (Fig. 1).

The oral feeding could be resumed two days after the surgery.

The postoperative course was uneventful (Dindo-Clavien classification II) and the patient was discharged 14 days after the surgery.

After an asymptomatic period of two months, mild hypoglycemias recurred, on average once every three weeks, easily managed by sugar ingestion. A preexisting gastroparesis was worsened by the intervention, possibly caused by vagus nerve injury secondary to the multiple gastric procedures. A year later, the patient returned to a regular lifestyle.

**Fig. 2:**
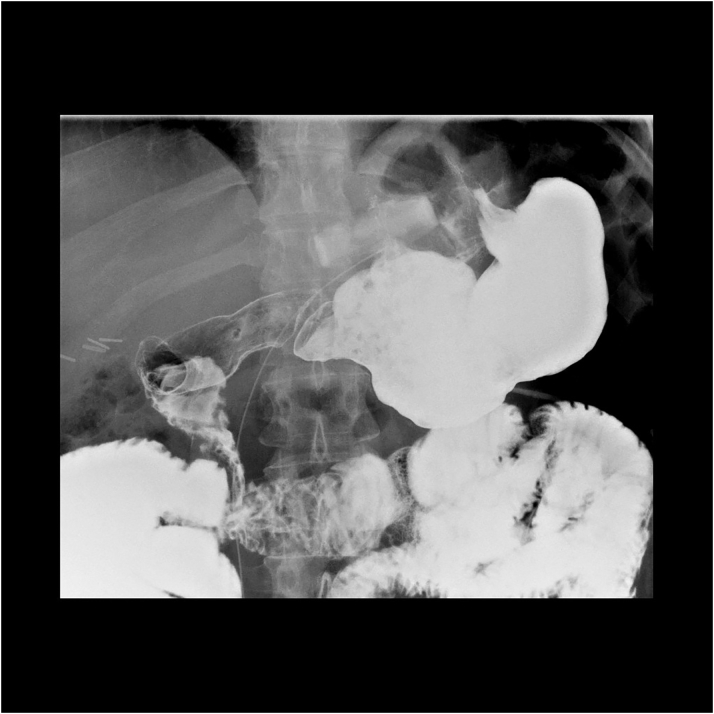
1-year postoperative upper GI series. The gastric banding is at the right spot and there is no stenosis nor leakage to be seen.

Her maximum weight was 105 kg (BMI: 37,6 kg/m^2^) and her weight prior to the RYGB was 98 kg (BMI: 35,1 kg/m^2^). After the latter surgery, the minimal weight reached by the patient was 68 kg (BMI: 24,4 kg/m^2^) and the weight right before the reversal and gastric banding was 70 kg (BMI: 25,1 kg/m^2^, excess weight loss: 100 %, total weight loss: 33,3 %). At 1-year follow-up, the weight regain was limited to 8kg and reached 78kg (BMI: 28 kg/m^2^) (Fig. 2).

## 3 Clinical discussion

Hypoglycemia is defined by the American Diabetes Association Workgroup on Hypoglycemia as a measured plasma glucose concentration ≤70 mg/dL (≤3.9 mmol/L) [6].

Sometimes, severe hyperinsulinemic hypoglycemias may lead to a potentially life-threatening central nervous system glucose deficiency called neuroglycopenia.

Originally described in pediatric congenital disorders by Laidlaw in 1938, the term nesidioblastosis refers to endogenous hyperinsulinemic hypoglycemia attributable to pancreatic cellular hyperfunction and hypertrophy [7].

In 2005, Service et al. were the first to describe postprandial symptoms of neuroglycopenia following RYGB related to nesidioblastosis confirmed in resected specimens from six patients who underwent partial pancreatectomy, with good results over the hypoglycemic symptoms [8].

Initial treatment includes strict low carbohydrate diet. Therapy with diazoxide, acarbose, calcium channel blockers and octreotide can be used after that, but their effect is highly unpredictable [9].

Differential diagnosis for post gastric bypass hypoglycemia includes late dumping syndrome, NIPHS and insulinoma. Late dumping syndrome, NIPHS and nesidioblastosis could have the same physiopathology with hyperfunction of beta cells causing hyperinsulinemic hypoglycemia, although different mechanisms, including GLP-1 and post-prandial insulin surge, may be involved [9].

After failure of hypoglycemic episodes management with diet and medication, surgery may become the best option. Mala et al. described an exhaustive list of all surgical procedures that have been performed and published, including gastric tube placement, reversal of the RYGB with or without concomitant sleeve resection, gastric pouch restriction and pancreatic resection [10]. Partial, sub-total, and total pancreatectomy have been performed to treat refractory hypoglycemia [11], [12], but the risk of iatrogenic diabetes currently makes them second-stage choices. Vanderveen et al., at the Mayo Clinic, also showed that 90 % of the patients who underwent partial pancreatectomy for noninsulinoma pancreatogenous hypoglycemia from diffuse islet cell disease have recurrent symptoms and 25 % of them no improvement in quality of life [13].

Several studies describe good results over severe postprandial hypoglycemias after RYGB reversal [14], [15], [16]. Even if the feasibility of the procedure is established, the weight regain after reversal remains a great concern.

Laparoscopic reversal of RYGB and placement of an adjustable gastric band after temporary gastrostomy in the gastric remnant has not yet been described in the literature for this indication. We think this could potentially help to overcome hypoglycemic episodes, while reducing weight regain often associated with obesity-related comorbidities.

## 4 Conclusion

This is the first case of NIPHS after RYGB treated with combined laparoscopic reversal of gastric bypass and gastric banding placement. We expect this approach will help to overcome NIPHS hypoglycemic episodes while maintaining weight control. Further studies with long term follow-up are necessary.

The following is the supplementary data related to this article.

Video S1 Combined laparoscopic reversal and gastric banding video. Video S1

## Provenance and peer review

Not commissioned, externally peer-reviewed.

## Sources of funding

None.

## Ethical approval

N/a.

## Consent

Written informed consent was obtained from the patient for publication of this case report and accompanying images. A copy of the written consent is available for review by the Editor-in-Chief of this journal on request.

## Author contribution

The authors contributed equally to the redaction of this case report and surgical video.

## Research registration

N/a.

## Guarantor

François-Xavier Terryn.

## Declaration of competing interest

None.
